# Hepatitis B virus integration and hepatocarcinogenesis

**DOI:** 10.1016/j.livres.2025.09.002

**Published:** 2025-09-11

**Authors:** Linlin Ma, Shuzhen Chen, Hongyang Wang, Lei Chen

**Affiliations:** a Fudan University Shanghai Cancer Center, Department of Oncology, Shanghai Medical College, Fudan University, Shanghai, China; b National Center for Liver Cancer, Naval Medical University, Shanghai, China; c International Cooperation Laboratory on Signal Transduction, Third Affiliated Hospital of Naval Medical University, Shanghai, China

**Keywords:** Hepatocellular carcinoma (HCC), Extrachromosomal circular DNA (ecDNA), Hepatitis B virus X (HBx) protein, Hepatocarcinogenesis, Integration

## Abstract

Hepatitis B virus (HBV) is the most common cause of hepatocellular carcinoma (HCC), which is the predominant liver cancer type in Southeast Asia. Approximately 350 million individuals suffer from persistent hepatitis B infection worldwide. HBV promotes HCC development through direct and indirect mechanisms. HBV DNA integrates into the host genome during the initial stages of tumorigenesis, causing insertional mutagenesis of cancer-related genes and genomic instability. Extrachromosomal circular DNA (ecDNA) is formed, which is efficiently amplified in large quantities to express viral genes and host oncogenes. Moreover, virus-associated proteins, such as the regulatory HBV X (HBx) protein and/or the modified preS/S envelope protein, alter the expression of genes associated with multiple functions in host cells. In this review, we summarize the role of the HBx and preS/S proteins in promoting tumorigenesis. In addition to summarizing the specific mechanism of HBV-related tumorigenesis, the concerns and perspectives for future study are discussed.

## 1 Introduction

Hepatocellular carcinoma (HCC) is the third most common cause of cancer-related deaths worldwide and the sixth most common type of cancer overall.^1^^,^^2^ Based on the latest estimates from the World Health Organization, the global seropositivity of hepatitis B surface antigen (HBsAg) is 3.8%. The global age-standardized death rate from hepatitis B virus (HBV)-related cirrhosis in 2019 was 4.03 per 100,000 individuals.^3^ Nearly half of the cirrhosis and chronic liver disease deaths and three-fourths of the HCC deaths occur in the Asia-Pacific region, which harbors the largest global burden of HBV infection. Of the 257.5 million people infected with HBV worldwide in 2022, approximately 65% live in the Asia-Pacific region. According to the Global Burden of Disease 2019 Study, an estimated 555,000 individuals worldwide died from HBV infection, of which 375,000 (67.5%) were in the Asia-Pacific region.^3^, ^4^, ^5^

HCC development is influenced by a combination of genetic predisposition, environmental factors (*e.g.*, metabolic syndrome, alcohol, aflatoxin B1, and aristolocholic acid), and viruses (*e.g.*, HBV and HCV). Chronic HBV infection accounts for at least half of all HCC cases worldwide. Based on epidemiological studies, HBV carriers are ten times more likely to develop HCC compared with noncarriers.^6^ Aflatoxin B1 (AFB1) exposure combined with chronic HBV infection results in an exceptionally high rate of HCC in subtropical Asian and African countries. Although preventative vaccines have become prevalent over the past decade, the statistics indicate that while immunization significantly lowers the risk of contracting hepatitis B, there is still a considerable number of individuals infected with hepatitis B because of mother-to-child transmission.^7^^,^^8^ Moreover, chronic hepatitis B (CHB) patients are still considered incurable and are at risk for developing HCC. Most liver cancers arise from cirrhosis; however, cirrhosis is absent in up to one-third of patients with HBV-related HCC.^9^^,^^10^ The possibility that HBV directly contributes to liver transformation is supported by the development of HBV-associated HCC in non-cirrhotic livers. Therefore, the underlying mechanisms responsible for HCC tumorigenesis related to HBV infection have remained an active area of investigation for decades.

HBV is an enveloped DNA virus consisting of three viral surface antigens that surround a nucleocapsid composed of hepatitis B core (HBc) protein, viral polymerase (Pol), and viral genomic DNA. Its genomic DNA exists in a relaxed circular DNA (rcDNA) form and is approximately 3.2 kb in length, with a complete minus strand and an incomplete plus strand. The HBV genome evolves relatively slowly and is highly conserved. Based on sequencing analyses of HBV genes from ancient cadavers dating back 800 years and 4500 years, the annual replacement mutation rate of each base was determined to be 8.04 × 10^−6^−1.51 × 10^−5^, which suggests that the tree root for HBV was established 8600–20,000 years ago.^11^^,^^12^

The viral genome encodes four open reading frames (ORFs), C, P, S, and X, from which functional viral proteins are produced. These include HBc from C, Pol from P, three types of surface antigens (preS1, preS2, and HBs) from S, and the HBV X (HBx) protein from X. Of these, HBx is a multifunctional protein that not only promotes virus production at multiple steps, including viral transcription and replication, but also plays a role in the development of HBV-related HCC. Moreover, the emergence and takeover of HBV variants carrying mutation(s) in the preS/S genomic region is a frequent event, which suggests an essential role for preS/S in hepatocarcinogenesis.^13^, ^14^, ^15^

## 2 The integration of the HBV genome into the host genome

HBV integration refers to the insertion of a fragment or whole HBV genome into the host genome. After the receptor-mediated entry of HBV into the cell, the nucleocapsid containing the rcDNA genome is released into the cytoplasm and moves to the nucleus. The released rcDNA is transformed into covalently closed circular DNA (cccDNA) through the activity of nuclear host cytokines, which are engaged in various aspects of DNA repair. The cccDNA is transcribed into a pre-genomic RNA (pgRNA) and messenger RNA. The pgRNA is encased in the viral capsid along with the viral polymerase. Reverse transcription of pgRNA occurs through a complex series of steps inside the nucleocapsid to produce rcDNA or double-stranded linear DNA (dslDNA).^16^^,^^17^ This dslDNA is an abnormal by-product of viral DNA synthesis caused by in situ primers of the viral DNA plus strand,^18^^,^^19^ which may be recycled into the nucleus or released from the liver cells as fresh virions.^17^ The dslDNA is usually inserted into the human genome through terminal direct repeat binding. Once integrated into the nucleus, dslDNA replication intermediates may be used as substrates for the integration of the host genome at broken double-stranded DNA sites through either non-homologous end ligation or microhomologous-mediated end ligation, which occurs in 1 out of 10^5^ to 10^6^ infected cells.^19^

Although the ORFs of the DNA polymerase and HBsAg/hepatitis B core antigen (HBcAg) are split off from their promoters in their integrated form, the HBsAg ORF is still expressed.^20^^,^^21^ Several studies indicate that integrated HBV DNA expresses only the surface, but not the core encoding transcript.^22^^,^^23^ Therefore, how the structure of the HBV DNA is rearranged during integration with the host genome influences the function of the ORF of the virus is unclear.^20^

## 3 HBV DNA integration is associated with HCC development

### 3.1 Clinical implications of HBV integration with HCC

HBV DNA integration into host chromosomes occurs in approximately 90% of HBV-related HCCs, and this integration can be observed at the early stages of HBV infection *in vitro* and *in vivo*.^19^^,^^24^, ^25^, ^26^, ^27^ Nucleos(t)ide analogues, which are known to inhibit HBV replication and decrease HBsAg levels, are commonly used for the treatment of HBV, thereby mitigating HCC risk. A study of 28 patients revealed that nucleos(t)ide analogues reduced HBV DNA integration (median integration frequency was 1.01 × 10^9^ before treatment and 5.74 × 10^8^ after 1 year of treatment). The median integration frequency after 10 years of treatment was 4.84 × 10^7^, and hepatocellular clonal expansion (from 2.41 × 10^5^ before treatment to 1.22 × 10^5^ after one year of treatment, and 2.55 × 10^4^ after 10 years of treatment) significantly decreased, suggesting that there is an alternative pathway to reducing HCC risk besides direct viral suppression.^28^ Currently, the focus of HBV antiviral therapy is to achieve a functional cure; that is, the elimination of HBsAg. Spatial transcriptome studies have revealed that transcriptionally active HBV integration is lower in HBsAg-deficient patients (almost undetectable), which provides further evidence that treatment with nucleos(t)ide analogues is effective at the long-term prevention of HCC.^29^

### 3.2 Development of HBV integration to HCC

It requires decades for HBV infection to result in HCC because the infected liver cells need to overcome many host defense barriers for transformation to occur. Once liver cells are infected with HBV, those carrying integrated HBV DNA are monitored, and most are eliminated by the host immune system; however, some liver cells with integrated HBV DNA are capable of evading immune clearance. When chronic HBV infection enters the inactive carrier phase, HBV DNA replication is restricted and evades immune surveillance. During this stage, the integrated HBV can still promote cell proliferation through the regulatory function of DNA insertional mutations. The CHB mouse model indicated that sustained recognition of HBsAg resulting from HBV integration rendered envelope-specific CD8^+^ T cells capable of sustaining prolonged moderate hepatocyte injury. Moderately severe, but persistent CD8^+^ T-cell-dependent liver disease underlies persistent hepatocellular necrosis and regeneration, which ultimately leads to the progression of CHB.^30^, ^31^, ^32^, ^33^ With the help of other concomitant somatic mutations, HBV DNA integration may enable individual cancerous hepatocytes to gain a clonal proliferative advantage and promote liver cancer.

Although HBV integration occurs randomly in chromosomes in CHB-infected liver tissues, there are HBV DNA integration hotspots in the chromosomes of HCC cells, such as telomerase reverse transcriptase (TERT), gene promoters, and exons of mixed-lineage leukemia-4 (MLL4), which are associated with HCC tumorigenesis. The emergence of these HBV integration hotspots suggests that HBV-integrated hepatocytes undergo positive selection through a specific mechanism.^34^^,^^35^ When irregular mutations accumulate in cells, some gain an advantage in uncontrolled proliferation and expand rapidly. Clonal expansion of error-carrying liver cells is common in HCC tissues, but occurs less frequently in normal liver tissues, although the emergence of positively selected clones is not necessarily associated with the presence of cancer. However, some studies suggest the presence of a common tumor driver gene in selected clones of normal tissues, which provides evidence for a strong link to cancer development during the early stages of carcinogenesis.^36^

Although HBV integration is common in tumor and nontumor hepatocytes, their patterns of integration differ. Compared with nontumor tissues, HCC exhibits a higher frequency and number of integration events in coding or promoter regions.^37^^,^^38^ More than three-quarters of tumor tissue exhibited HBV integration, whereas nontumor tissue contained less than half of that amount.^39^ Next-generation sequencing studies have also indicated significant differences in HBV integration patterns between tumor and adjacent nontumor tissue samples from the same patient.^37^^,^^40^^,^^41^ Of the several hundred HBV insertions detected in tumor and nontumor tissues, only 64 genes were identified as shared.^37^ Other than the discrepancy in mutation number and pattern, a relatively random distribution of integration sites was observed in nontumor samples; however, the integration sites in tumor samples were enriched on chromosomes 5, 16, 17, and 19, indicating preferential integration into chromosomes.^42^ Similarly, while HBV integration is very common in CHB, only a minority of the viral integration events are pathogenic. Several factors may result in the different outcomes for HBV carriers, such as viral DNA integration pattern, gene mutation caused by the integration, and genetic heterogeneity of HBV carriers.^43^

## 4 Mechanisms underlying HBV integration-induced HCC

Studies have shown that, in general, integrated HBV DNA does not produce pgRNA or facilitate HBV replication.^17^ This has led to theories that integrated HBV DNA is a cause of carcinogenesis. The reported mechanisms include (i) Insertion mutations, in which viral DNA is integrated into host genes, such as TERT, cyclin E1 (CCNE1), and MLL4, thus promoting genomic instability; (ii) Unstable chromosomes caused by incorporated HBV DNA;^44^ and (iii) Viral oncoproteins, including HBx and S/preS, which alter cell function and trigger carcinogenic pathways. Studies of human HBV-associated HCC have revealed high heterogeneity of the driving mechanism (Table 1).^37^^,^^42^^,^^45^, ^46^, ^47^, ^48^, ^49^, ^50^, ^51^

**Table 1:** HBV genome integration induces insertional mutagenesis and chromosomal instability.

Mechanisms	Region	Targeted gene	Tumor *vs*. adjacent liver	Function	Reference
Insertional mutagenesis	Near the coding genes	*TERT* gene (23.7%)	High in tumors Tumor: (86.4%) adjacent liver: (30.7%)	Inducing telomerase transcription and promoting resistance to cell aging and death in tumors	^37^^,^^45^^,^^46^
		*MLL4* gene (11.8%)		Downregulating the p53 tumor suppressor pathway	^37^^,^^47^^,^^48^
		*CCNE1* gene (5%)		Inhibiting the RB pathway, affecting the G1/S cell cycle	^37^
	Inside or close to repetitive noncoding sequences	HBx-LINE1	Unknown	Inhibiting miR-122, promoting EMT-like alterations, and inhibiting autophagy	^42^^,^^49^
				Affecting Wnt/β-catenin pathway	^42^^,^^50^
Chromosomal instability	Mainly in the intergenic regions and repetitive sequences	A set of caspase genes	High in tumors (somatic copy number variation positively correlated with HBV integration)	Loss of copy numbers at the caspase site leads to the inhibition of apoptosis.	^42^^,^^51^

Abbreviations: CCNE1, cyclin E1; EMT, epithelial mesenchymal transformation; HBV, hepatitis B virus; HBx-LINE1, hybrid RNA transcript of the human LINE1 and the HBV-encoded X gene; miR, microRNA; MLL4, mixed-lineage leukemia-4; RB, retinoblastoma; TERT, telomerase reverse transcriptase.

### 4.1 Insertional mutagenesis

HBV integration appears to exert insertional mutation pressure on the human genome. Approximately 40% of the HBV breakpoints in the HBV genome occur near viral enhancers, *X* genes, and core ORFs, whereas the majority of HBV breakpoints are located near coding genes, primarily in exons or regulatory regions of genes, such as *TERT*, *MLL4*, and *CCNE1*.^37^ Frequent insertion of HBV DNA into *TERT* promoters induces TERT expression and promotes resistance to cell aging and death.^45^^,^^46^ Recent studies indicate that HBV also inserts into the intron region of *TERT*, which results in increased *TERT* expression in HCC; however, the specific regulatory mechanism of HBV intron insertion leading to gene upregulation is unclear.

The HBV integration breakpoint is frequently inserted into *MLL4* either in the intron or exon.^37^^,^^48^ MLL4 is a member of the ASC-2 complex and a histone lysine N-methyltransferase that functions in the p53 tumor suppressor pathway. HBV insertions into *MLL4* often result in epigenomic modification.^47^^,^^52^ Transcriptome profiling data indicate a more than 20-fold increase in MLL4 expression in HCC. The recurrence of *MLL4* gene integration sites demonstrates the pathogenic role of HBV integration in HCC.^37^

In eukaryotic cells, cyclins are known to be associated with carcinogenic signals and are primarily engaged in regulating cell cycle events. The *CCNE1* gene is also a common target for recurrent HBV integration in patients with liver cancer.^37^ Recurrent HBV insertions into *CCNE1* inhibit the retinoblastoma (RB) pathway, which regulates the G1/S cell cycle.^37^ RNA sequencing analysis confirmed that the HBV gene fuses with the last exon of *CCNE1* to form transcripts, which results in an increase in cyclin E1 expression and is associated with reduced disease-free survival.^53^ Therefore, HBV integration into the *CCNE1* gene locus drives abnormal cell cycle regulation during the emergence and progression of HCC.

HBV DNA integration also occurs inside or close to repetitive noncoding sequences, which consist of long interspersed nuclear elements (LINEs) and short interspersed nuclear elements (SINEs).^42^^,^^54^ The virus–human hybrid HBx-LINE transcript was discovered by Lau *et al*.^55^ in HBV-positive HCC, which exhibits carcinogenic qualities independent of its protein product and is associated with reduced overall survival in HCC patients.^55^^,^^56^ HBx-LINE1 contains six miR-122 binding sites and serves as a molecular sponge for cellular miR-122, thus preventing HBV replication by interacting with cyclin G1.^57^^,^^58^ HBx-LINE1 inhibits miR-122, promotes epithelial mesenchymal transformation (EMT)-like alterations, and inhibits autophagy in cultured hepatic tumor cells.^49^ It also increases phosphorylated β-catenin levels, which is linked to cell proliferation and is an indicator of liver tissue damage.^50^ However, the HBV-LINE1 fusion transcript appears to be restricted to HBV genotype C in Asian population and has not been identified in HBV-associated HCC in European patients.^59^

Extrachromosomal circular DNA (ecDNA) is a type of circular DNA that exists outside of the chromosome and promotes carcinogenesis through multiple mechanisms. First, ecDNA amplifies oncogene expression through copy number gain. Because of the absence of centromeres, ecDNA is asymmetrically distributed during cell division, resulting in its unequal inheritance by daughter cells. This results in a subset of progeny cells harboring higher ecDNA copy numbers compared with those in the parental cells, thus enabling ecDNA-positive cancer cells to rapidly proliferate and accumulate through clonal expansion.^60^^,^^61^ Moreover, ecDNA augments transcriptional activity by increasing chromatin accessibility. In colorectal cancer and glioblastoma, ecDNA is organized into nucleosomes, but lacks higher-order compaction, which is a hallmark of transcriptionally repressive chromatin, thus potentiating an open chromatin state. Moreover, ecDNA exhibits reduced DNA methylation at regulatory elements, further destabilizing epigenetic silencing.^60^^,^^62^ Furthermore, ecDNA serves as a reservoir for DNA recombination, in which it promotes resistance to chemotherapeutic drugs and targeted therapies. Finally, circular ecDNA is resistant to digestion by exonuclease enzymes compared with linear DNA, so it persists long after it is released from cells into the bloodstream.^60^^,^^63^, ^64^, ^65^, ^66^, ^67^

**Fig. 1:**
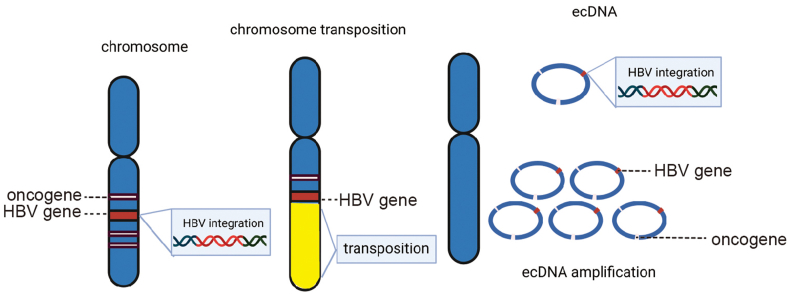
**The process of hepatitis B virus (HBV) integration leading to hepatocarcinogenesis.** Left: HBV is integrated into the human genome. Middle: HBV integrates into host chromosomes, undergoes chromosomal translocation, and activates host proto-oncogenes. Right: HBV-oncogene integration cyclizes into extrachromosomal circular DNA (ecDNA). The stable circular structure of ecDNA results in higher levels of oncogene transcription.

In HBV-related HCC, HBV-TERT integration cyclizes into ecDNA. The resulting HBV-oncogene-ecDNA structure increases HBV copy number and promotes oncogene expression (Fig. 1).^60^^,^^64^ We reported ecDNA in 27.3% of liver cancer samples and identified a total of 76 oncogenes in ecDNA, including proto-oncogenes, such as *MYC*.^64^ Because of the carcinogenic effects of ecDNA and their relatively stable characteristics in the bloodstream, ecDNA detection by sequencing yields possible clues for the evaluation of HCC prognosis.

### 4.2 Induction of chromosomal instability by the integrated DNA

Compared with cancers associated with other risk factors, HBV-related malignancies typically have a higher frequency of chromosomal abnormalities because of genomic instability through viral DNA integration.^68^^,^^69^ Increased copy number variations at HBV breakpoint positions were detected by whole-genome sequencing of HBV-related HCCs.^37^^,^^70^ Insertion of the HBV genome results in copy number alterations and genomic instability, which indirectly affects gene expression. One study showed that 10% of liver cancer samples exhibited a loss of copy number at the caspase site. This resulted in the loss of heterozygosity of a set of caspases (CASP1, CASP4, CASP5, CASP12) and caspase recruitment domain family members (CARD16 and CARD17), which further affected the execution stage of apoptosis. Some reports suggest that chromosome instability caused by HBV results in the augmentation of HBV integration in host cell lines,^71^^,^^72^ because DNA damage may cause more double-stranded DNA breaks, which provides an opportunity for HBV integration.

### 4.3 Role of viral proteins in carcinogenesis

**Fig. 2:**
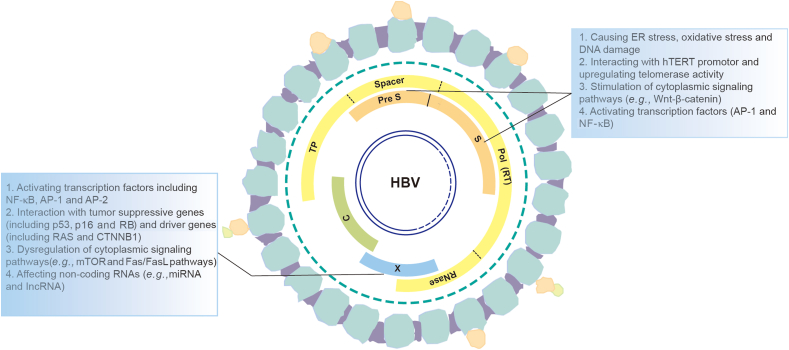
**The hepatitis B virus (HBV) genome DNA exists as a relaxed-circular DNA (rcDNA) that encodes four open reading frames (ORFs), C, P, S, and X.** X and preS/S are translated into the HBx protein and three surface antigens, respectively. HBx and PreS/S affect the etiology of HCC through various mechanisms. Abbreviations: AP, activator protein; CTNNB1, beta-catenin; ER, endoplasmic reticulum; hTERT, human telomerase reverse transcriptase; lncRNA, long-stranded noncoding RNA; miRNA, microRNA; mTOR, mammalian target of rapamycin; NF-κ B, nuclear factor-kappa B; RB, retinoblastoma.

Although HBV integration exhibits a relatively muted effect on viral replication, the exogenous expression of wild-type and truncated HBx or preS has been reported as the potential mediator for cell function, such as activating carcinogenic pathways (Fig. 2).^17^^,^^73^

#### 4.3.1 HBx

The *HBx* gene is the most frequently observed viral ORF that is incorporated into the host genome of individuals with liver cancer.^74^ *HBx* consists of two functional domains: COOH-terminal polypeptides (pro-apoptotic effects) and NH2-terminal intermediate peptides (carcinogenic effects). HCC develops as a result of the imbalance being disrupted, and carcinogenic pathways take over when the pro-apoptotic domain is eliminated by an unidentified mechanism. The HBx protein is a multifunctional protein that promotes liver cancer by (i) activating a variety of transcription factors; (ii) interacting with oncogenes and tumor suppressor genes; (iii) stimulating cytoplasmic signaling pathways; and (iv) interacting with noncoding RNAs (Table 2).^75^, ^76^, ^77^, ^78^, ^79^, ^80^, ^81^, ^82^, ^83^, ^84^, ^85^, ^86^, ^87^

**Table 2:** Carcinogenic mechanisms of HBx.

Mechanisms	Function	Source of evidence	Reference
Activating a variety of transcription factors	Promoting NF-κB to inhibit TNF-α and Fas-mediated apoptosis	Cell experiments (hepatoma cell lines: HepG2 and Huh7)	^75^

	Stimulating the transcription factors CREB and ATF-2	Cell experiments (hepatoma cell line: HepG2)	^76^

	Disrupting the Smc5/6 complex and relieving transcriptional repression	Cell experiments (hepatoma cell lines: HepG2 and HepAD38)	^77^
		+ animal experiments (human liver chimeric uPA-SCID mice)	
Interacting with oncogenes and tumor suppressor genes	Inhibiting tumor suppressive genes (inhibiting *p53* and inactivating the *RB* gene)	Cell experiments (hepatocyte cell line: THLE-5b; hepatoma cell lines: Hep3B, SK-Hep-1, HepG2, Huh7, 97L, PLC/PRF/5, and SMMC7721)	78, 79, 80
		+ animal experiments (HBx-transgenic mice and Balb/c nude mice)	
		+ clinical samples (paired HCC samples, *n* = 51)	
	Activating oncogenes: the Ras-GTP complex	Cell experiments	^81^
Stimulating cytoplasmic signaling pathways	Activating the mTOR signaling pathway	Cell experiments (hepatoma cell lines: HepG2, Hep3B, Huh7, and Hep2.2.15)	^82^
		+ animal experiments (DEN-induced HCC mouse model and spontaneous HCC mouse model)	
		+ clinical samples (paired HCC samples, *n* = 80)	
	Affecting the Fas/FasL signaling pathway	Cell experiments (hepatoma cell lines: HepG2, SNU-354, SNU-368, SNU-387, SNU-398, and SNU-423)	^83^^,^^84^
		+ animal experiments (HBx homozygote transgenic mice)	
		+ clinical samples (paired HCC samples, *n* = 15)	
Interacting with noncoding RNAs	Affecting miRNAs, such as miR-122 and miR-152	Cell experiments (hepatocyte cell line: LO2; hepatoma cell lines: Hepa1-6, HepG2, HepG2.2.15, and Huh7)	^85^^,^^86^
		+ animal experiments (HBx-transgenic mice)	
		+ clinical samples (chronic hepatitis B samples, *n* = 22; severe chronic hepatitis B samples, *n* = 19; normal liver tissues, *n* = 10; paired HCC samples, *n* = 20)	
	Affecting lncRNA, such as lncRNA DLEU2 and LINC01431X	Cell experiments (hepatoma cell lines: HepG2, HLCZ01, HepAD38, Huh7, and HepG2.2.15)	^87^
		+ animal experiments (hydrodynamic injection mouse model)	
		+ clinical samples (para-tumor tissues, *n* = 41)	

Abbreviations: ATF-2, activating transcription factor-2; CREB, cAMP response element binding protein; DEN, diethylnitrosamine; HBx, hepatitis B virus X; HCC, hepatocellular carcinoma; lncRNA, long-stranded noncoding RNA; miRNA, microRNA; mTOR, mammalian target of rapamycin; NF-κB, nuclear factor-kappa B; RB, retinoblastoma; Smc5/6, structural maintenance of chromosomes 5/6; TNF-α, tumor necrosis factor-alpha.

Several transcription factors, including nuclear factor-kappa B (NF-κB), activator protein-1 (AP-1), and activator protein-2 (AP-2), are transactivated by HBx. In addition, the expression of the protein kinase C (PKC) activator diacylglycerol (DAG) is induced by HBx, which increases the expression of PKC-dependent transcription factors, thereby promoting HBV replication and the occurrence of HCC. Another study reported that HBx enters the protein-protein complex of the cellular transcription factors cAMP response element binding protein (CREB) and activating transcription factor-2 (ATF-2), resulting in the binding of the complex to HBV enhancers, which in turn, increase HBV replication and promote hepatocarcinogenesis.^76^ A recent study demonstrated that the structural maintenance of chromosomes 5/6 (Smc5/6) complex plays a role as a restriction factor that specifically prevents ecDNA transcription. HBx protein enhanced Smc5/6 transcriptional suppressor complex ubiquitination, thus stimulating the transcription of HBV cccDNA.^77^

HBx promotes hepatocarcinogenesis by interacting with tumor suppressor genes (*e.g.*, *p16*, *p53*, and *RB*) or oncogenes (*e.g.*, *RAS* and *CTNNB1*). Tumor suppressor gene promoters, such as *p16/INK4A*, become regionally hypermethylated through the relocation of DNA methyltransferase 3A (DNMT3A) when bound by HBx.^88^ HBx binds to TP53 and directly inhibits p53 nucleotide excision repair and transcription-coupled repair, weakening p53-mediated apoptosis and cell cycle checkpoint function.^78^ Furthermore, HBx indirectly reduces p53 expression by downregulating two p53 activators, apoptosis-stimulating p53 proteins 1 and 2 (ASPP1 and ASPP2).^79^ Moreover, HBx interacts with the p53 regulator prolyl isomerase NIMA-interacting 1 (Pin1), which activates downstream target genes and promotes tumorigenesis. Through promoter methylation, HBx suppresses cyclin-dependent kinase (CDK) inhibitors, which inactivates the RB tumor suppressor.^80^

Besides inhibiting tumor suppressive genes, HBx activates oncogenes through various mechanisms. Ras, a well-known proto-oncogene, is associated with multiple cancers when abnormally activated. The Ras protein is a monomeric GTPase that may be activated and promote cell proliferation after binding GTP to form a Ras-GTP complex. HBx enhances GTP absorption, while keeping Ras-specific GTPase-activating protein (Ras-GAP) unchanged.^81^ However, HBx acts indirectly on upstream activators of Ras (*e.g.*, Shc, Grb2, and SOS) and promotes the synthesis of Ras-GTP.^89^ HBx also stimulates Ras through the Src family of tyrosine kinases and induces transcriptional transactivation through AP-1.^89^ The stability of CTNNB1 is also dysregulated by HBx-mediated degradation of GSK3β.^90^^,^^91^

Numerous reports indicate a role for HBx in altering cytoplasmic signaling pathways. HBx binds to the asparagine synthetase (*ASNS*) promoter, resulting in a concomitant increase in asparagine (Asn) levels. Increased Asn activates the mTOR pathway and promotes tumor cell proliferation.^82^ Bid is a proapoptotic member of the Bcl-2 family. Reduced Bid expression and increased interleukin-18 (IL-18) level render HCC cells resistant to Fas-induced apoptosis, which is associated with HBx.^83^^,^^84^ Moreover, HBx increases calcium entry through enhanced mitochondrial calcium uptake and storage, resulting in high cytoplasmic calcium and reactive oxygen species (ROS) levels.^92^ By regulating the activation of calcium-mediated signaling proteins, such as the CAMKK/CAMKIV pathway, the secretion of high-mobility group box 1 (HMGB1) from HCC cells is induced, thus promoting the proliferation and metastasis of tumor cells.^93^

Noncoding RNAs, such as microRNAs (miRNAs) and long-stranded noncoding RNAs (lncRNAs), have an important role in hepatocarcinogenesis and progression. The expression of several miRNAs is altered in HCC, such as cancer-promoting miRNA (miR-224) and cancer-inhibiting miRNA (*e.g.*, let-7 family members, miR-122, miR-152, and miR-26a).^94^, ^95^, ^96^ HBx binds to specific transcription factors to inhibit let-7 transcription and interacts with certain cytokines to interfere with its maturation, thus downregulating let-7. HBx also downregulates miR-26a and activates the tumor-promoting Wnt/β-catenin pathway. In addition, the function of miR-122 in preventing HBV replication may be inhibited by HBx.^57^^,^^85^ Moreover, HBx may inhibit the function of miR-224 and miR-152, thus facilitating the occurrence of liver cancer.^86^^,^^97^ In addition to miRNAs, HBx affects the expression and function of lncRNAs, such as DLEU2 and LINC01431,^87^^,^^98^ which are involved in regulating the transcription of viral cccDNA.

The HBx protein interferes with host immune responses through multiple mechanisms, including the suppression of antiviral responses, increased immune tolerance, and establishing a tumor microenvironment. For example, HBx activates the NF-κB signaling pathway, which upregulates the expression of inflammatory cytokines, such as IL-10, leading to immunopathological damage.^99^ Moreover, HBx downregulates the stability of mitochondrial antiviral signaling protein, which inhibits interferon (IFN) production and disrupts the innate immune response.^100^ HBx has been shown to upregulate the expression of Fas and FasL *in vitro*, which enables immune surveillance evasion and promotes tumorigenesis.^101^ Collectively, HBx modulates immune-related cytokines and induces immune tolerance, thereby establishing an immunosuppressive microenvironment that promotes tumor survival.

C-terminal truncated HBx (Ct-HBx) proteins have been routinely detected in HCC tumor tissues compared with nontumor tissues.^102^^,^^103^ Compared with full-length HBx, Ct-HBx has a more pronounced effect on growth-inhibiting miRNAs, which promotes hepatocyte proliferation.^104^ HBxΔ35 is a variant of HBx with 35 amino acids missing from the C-terminus. HBxΔ35 regulates cell growth by directly silencing growth arrest-specific protein 2 (GAS2), which induces p53-dependent apoptosis and senescence, thereby conferring a survival advantage to precancerous hepatocytes and promoting cancer development.^105^

#### 4.3.2 HBsAg and PreS/S

HBV integration markedly increases HBsAg levels in CHB patients. HBsAg transcriptional activity in integrated HBV DNA increases considerably, particularly in HBeAg-negative patients.^106^, ^107^, ^108^, ^109^ A significant risk of HCC has been linked to the presence of preS mutations in serum.^110^, ^111^, ^112^ The PreS/S ORF of HBV contains three translation initiation sites, PreS1, PreS2, and S, which are translated into the large HBV surface protein (LHBst), middle HBV surface protein (MHBst), and small HBV surface protein (SHBst). The *S* gene region is stable, whereas the PreS1 and PreS2 regions are unstable and prone to mutation.^113^ Truncated S gene mutants exhibiting partial deletions in their preS regions induce endoplasmic reticulum (ER) stress, oxidative stress, and DNA damage.^110^^,^^114^, ^115^, ^116^ Eventually, excess oxidative substances act on oncogenes or certain tumor suppressor proteins.^117^^,^^118^

Trans-activators encoded by integrated 3′-truncated preS/S increase the expression of some genes involved in liver cell proliferation.^119^ The 3′-terminated PreS/S sequences can generate functionally active C-terminally truncated MHBst.^120^ MHBst proteins act as tumor promoters by activating key enzymes associated with proliferation, such as the PKC isoforms α/β or c-Raf-1/Erk2.^121^ In addition, MHBst directly interacts with the hTERT promoter's DNA response region, upregulating telomerase activity and accelerating HCC development.^122^ It can also cooperate to activate the Wnt/β-catenin signaling pathway to promote HBV-integrated hepatocyte replication and promote malignant transformation.^123^ Similar to MHBst, LHBst activates a variety of transcription factors, including AP-1 and NF-κB, in PKC-dependent manner.^124^

PreS proteins also play an important role in shaping the tumor immune microenvironment. The PreS protein can significantly enhance the immune response to anti-HBs;^125^ however, the PreS1 and PreS2 regions are inherently unstable and prone to mutation. Such mutations may result in the defective secretion of viral proteins and particles, and the accumulation of HBsAg in the ER. This subsequently induces ER stress, promotes increased ROS production, genomic instability, and ultimately, hepatocarcinogenesis. Furthermore, the PreS1, PreS2, and S domains contain multiple B-cell and T-cell epitopes. Deleting or altering these epitopes may enable mutant viruses to evade immune clearance.^126^

## 5 Diagnosis and treatment

HBV integration represents a novel cell-free tumor DNA biomarker, virus-host chimera DNA (vh-DNA). Vh-DNA is a unique DNA fragment generated from the junction of HBV integration into HCC chromosomes, which is detectable in blood using droplet digital PCR (ddPCR). In HCC tumors >1.5 cm, the signature junctional vh-DNA fragment can be detected in blood, and its abundance strongly correlates with tumor size, suggesting its potential diagnostic utility. Furthermore, postoperative vh-DNA levels are associated with residual tumor cells and may serve as an independent risk factor for predicting early recurrence. Analysis of circulating vh-DNA during recurrence can determine the clonal origin of the tumor, whether it arises from de novo formation or true recurrence.^127^^,^^128^

Several advances have been made in the treatment of HBV-associated HCC. First, the heterogeneity of HBV integration sites in the host genome leads to diverse oncogenic pathways in HCC. Consequently, variability in the HBV integration site may result in distinct prognostic outcomes and differential therapeutic responses to immune checkpoint inhibitors or targeted therapies (including anti-angiogenic agents and multi-kinase inhibitors). This suggests the potential for precision medicine approaches tailored to individual patients based on their specific HBV integration profiles.^129^ Similarly, targeted nucleic acid therapies designed to suppress the expression of these integration-associated genes represent a novel approach for establishing subgroup-specific treatments for HCC.^128^^,^^130^ Finally, the integration of HBV sequences into the host genome generates chimeric antigens, whose distinct molecular signatures may facilitate the development of therapeutic vaccines for HBV-induced HCC or enable the targeted elimination of hepatocytes expressing HBV-derived chimeric antigens through antigen-specific T-cell therapies.^131^^,^^132^

## 6 Conclusions and perspectives

HBV is a significant risk factor for HCC worldwide. HBV DNA integrates into the host genome during the initial stages of clonal tumor growth, causing genomic instability and the direct insertional mutagenesis of various cancer-related genes. HBV DNA integrates into the host genome for rapid replication and utilizes a circular structure of ecDNA to amplify itself in large quantities, thus increasing the copy number of certain oncogenes. Abnormal expression of viral regulatory protein HBx, as well as modified preS/S envelope proteins, facilitates the malignant transformation of HCC. HBx promotes liver cancer development by activating various transcription factors and noncoding RNAs that interact with oncogenes and tumor suppressor genes and dysregulate cytoplasmic signal transduction pathways. PreS/S envelope proteins influence transcription by activating gene promoters through a variety of mechanisms.

Although many studies have been conducted on HBV integration, the precise mechanism of HBV integration into the host genome, its function in viral replication, and its clinical significance remain unclear. The following issues need to be addressed in future studies: (i) HBV integration exists not only in chromosomal genomes, but also in ecDNA. How is HBV DNA integrated into ecDNA, and what is the implication for virus elimination in the clinic? (ii) How are target genes selected for HBV integration, and what is the exact mechanism? (iii) Because HBV integration sites vary within different individuals, how can we develop effective treatment based on different HBV integration sites for precision medicine?

Along with advances in gene sequencing, the enhanced reads and resolution have greatly expanded our understanding of HBV integration. Wang *et al*.^133^ combined the Chromium 10X Genomics approach and the full-length Smart-seq2 tablet-based approach, which are two RNA-seq platforms for multi-tissue analysis of liver cancer samples and provide a platform for large-scale data integration and in-depth analyses.^133^^,^^134^ Our group has also conducted deep whole-genome sequencing of HCC, which details the complex evolutionary history and genetic landscape of HCC in the Chinese population.^64^ These methods provide novel perspectives for understanding the evolution of HBV-associated pathogenesis and may shed light on innovative therapeutic strategies and treatments.

## Authors’ contributions

**Linlin Ma:** Writing – review & editing, Writing – original draft, Visualization. **Shuzhen Chen:** Writing – review & editing, Writing – original draft, Funding acquisition. **Hongyang Wang:** Conceptualization, Supervision, Project administration, Writing – review & editing, Funding acquisition. **Lei Chen:** Conceptualization, Supervision, Project administration, Writing – review & editing, Funding acquisition.

## Declaration of competing interest

The authors declare that there is no conflict of interest.
